# Biomimetic proteolipid vesicles delivering small activating RNA to activate the macrophage immunotherapy for the treatment of lung cancer

**DOI:** 10.1186/s12951-026-04392-4

**Published:** 2026-04-16

**Authors:** Hui Yu, Jiandong Zhang, Qiuyun Liu, Ling Liu, Yingying Le, Huiyu Cen, Weijie Peng, Juyan Wei, Sijia Liu, Aiping Qin, Yuyan Qin, Xiufeng Li, XiaoGang Xu, Lingmin Zhang, Lu Liang

**Affiliations:** 1 The Fifth Affiliated Hospital, Key Laboratory of Molecular Target & Clinical Pharmacology, State Key Laboratory of Respiratory Disease, The School of Pharmaceutical Sciences, NMPA, Guangzhou Medical University, Guangzhou, China; 2 State Key Laboratory of New Targets Discovery and Drug Development for Major Diseases, Gannan Innovation and Translational Medicine Research Institute, Gannan Medical University, Ganzhou, China; 3 Hutchison Whampoa Guangzhou Baiyunshan Chinese Medicine Co., Ltd, Guangzhou, China; 4 Center for Rehabilitation Medicine, Department of Ophthalmology, Affiliated People’s Hospital, Zhejiang Provincial People’s Hospital, Hangzhou Medical College, Hangzhou, Zhejiang China; 5 Department of Anesthesiology, The First Affiliated Hospital of Chongqing Medical University, Chongqing, China; 6 School of Basic Medical Sciences, Southern Medical University, Guangzhou, China

**Keywords:** Small activating RNA, Nanoparticles, Tumor-associated macrophages, Tumor microenvironment

## Abstract

**Background:**

Tumor-associated macrophages (TAMs) in the tumor microenvironment (TME) typically polarize toward an M2 phenotype that promotes tumor progression and immune suppression. Reprogramming TAMs to the proinflammatory M1 phenotype has emerged as a promising strategy to boost antitumor immunity. This study aimed to develop a targeted nanoplatform to deliver small activating RNAs (saRNAs) that upregulate genes involved in macrophage reprogramming.

**Results:**

We designed saRNAs targeting the promoters of p38 and TFEB, encapsulated within a metal-organic framework (MOF)-based delivery system and cloaked with hybrid membranes composed of synthetic lipids and exosome-derived vesicles. These were further functionalized with the TAM-targeting peptide CRV (CRVLRSGSC), resulting in nanoparticles termed CLMSR. CLMSR selectively accumulated in macrophages, enhancing intracellular saRNA delivery. Functional assays revealed that conditioned medium from CLMSR-treated M2 macrophages suppressed tumor cell migration, invasion, and 3D spheroid formation. In vivo, CLMSR demonstrated prolonged circulation time and enhanced tumor targeting. Importantly, treatment remodeled the TME by increasing CD8⁺ and CD4⁺ T cell infiltration and promoting TAM repolarization from the M2 to M1 phenotype.

**Conclusions:**

Our findings demonstrate that CLMSR represents a novel and efficient nanoplatform for saRNA delivery to reprogram TAMs and modulate the TME. By targeting M2 macrophages and inducing their transition to the tumoricidal M1 phenotype, this approach offers a promising therapeutic avenue to enhance antitumor immunity and inhibit tumor progression.

**Graphical abstract:**

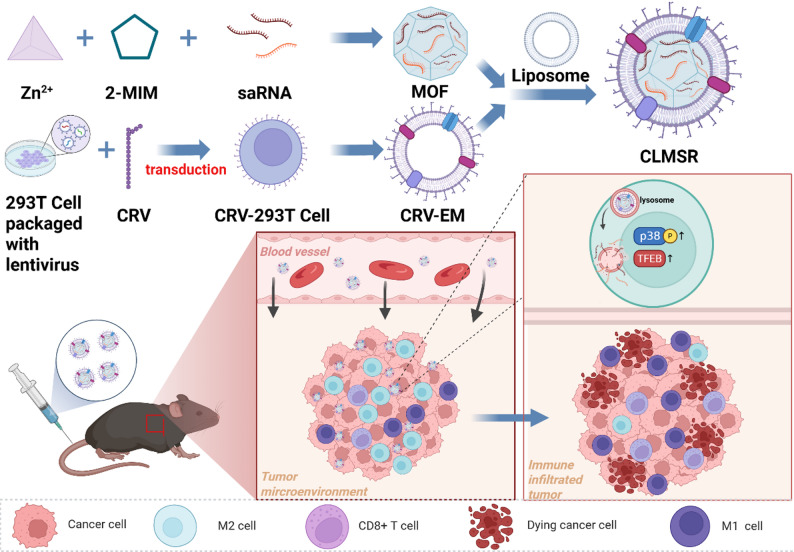

**Supplementary Information:**

The online version contains supplementary material available at 10.1186/s12951-026-04392-4.

## Background

Lung cancer has become one of the most fatal cancers in the world, and its 5-year survival rate is lower than 15%. The T cell-based immunotherapy, such as immune checkpoint blockage, chimeric antigen receptor T-Cell (CAR-T) therapy, or T-cell receptor engineered T cell therapy (TCR-T) has emerged as a promising approach for lung cancer therapy. However, these approaches are still challenged by the difficulties including T cell depletion and infiltration to the tumor environments (TME). Moreover, the tumor-promoting TME may re-educate the T cells and reduce the T cell killing on the lung cancer cells.

Tumor-associated macrophages (TAMs) are a key component of the TME, consisting of two primary subtypes with opposing roles: classically activated M1 macrophages and alternatively activated M2 macrophages [1]. M1 macrophages are known for their anti-tumor activities, including direct cytotoxic effects and antibody-dependent cell-mediated cytotoxicity (ADCC) that target tumor cells [2]. In contrast, M2 macrophages facilitate tumor progression by promoting tumor growth and metastasis, suppressing T cell-mediated immune responses, and enhancing angiogenesis [3]. Notably, these macrophage subtypes exhibit significant plasticity, allowing them to transition between M1 and M2 states in response to changes in the tumor microenvironment or therapeutic interventions. Understanding the multifaceted roles of TAMs in immunotherapy is crucial for gaining deeper insights into the tumor microenvironment (TME) [4, 5]. Additionally, targeting TAMs is becoming an increasingly attractive area of research, with the potential to enhance the effectiveness of existing immunotherapies by creating synergistic effects.

Recent studies have demonstrated that pharmacological reprogramming of TAMs from the M2 to the M1 phenotype represents a promising strategy to alleviate immunosuppression within the TME and enhance both innate and adaptive antitumor immunity. For example, chloroquine (CQ), a clinically approved antimalarial drug, has been reported to function as an immunomodulatory agent capable of resetting TAMs toward an M1-like phenotype [3]. Mechanistically, CQ elevates lysosomal pH in macrophages, leading to Ca²⁺ release through the lysosomal Ca²⁺ channel mucolipin-1 (MCOLN1). The released Ca²⁺ subsequently activates p38 MAPK and NF-κB signaling pathways, driving M1 polarization. In parallel, Ca²⁺-dependent activation of transcription factor EB (TFEB) reprograms macrophage metabolism from oxidative phosphorylation toward glycolysis, further stabilizing the M1 phenotype and improving antitumor immune responses [3]. Despite these encouraging findings, small-molecule–based TAM reprogramming strategies such as CQ suffer from inherent limitations, including lack of cell-type specificity, pleiotropic pharmacological effects, and the potential for systemic toxicity due to indiscriminate modulation of lysosomal function in non-target tissues.

saRNA, a type of double-stranded RNA, activates the expression of target genes, offering a potential solution for conditions that lack effective treatments [6–9]. Harnessing small activating RNA (saRNA) presents a promising avenue for developing novel therapeutics to address currently untreatable diseases. The concept of saRNA was first explored in 2006 when researchers demonstrated its ability to target gene promoters such as p21, VEGF, and E-cadherin, leading to transcriptional activation [10]. Since its inception, significant advancements in saRNA design, synthetic chemistry, and our understanding of biological mechanisms have paved the way for the practical application of saRNA [11] For instance, MiNA Therapeutics Ltd recently conducted a groundbreaking clinical trial utilizing RNA activation (RNAa) in advanced hepatocellular carcinoma patients, showing promising outcomes. Previous studies have shown that saRNAs can restore the expression of tumor suppressor genes such as PTEN in non-small cell lung cancer (NSCLC), reversing resistance to tyrosine kinase inhibitors (TKIs) in vitro [12]. Functional saRNAs upregulating PTEN expression in H-157 NSCLC cells reduced cell proliferation and increased apoptosis under TKI treatment, demonstrating a direct anti-tumor effect through gene activation [12]. These features render saRNAs particularly suited for lung cancer therapy, where restoration of tumor suppressor function and overcoming drug resistance are critical. Although saRNAs have significant advantages, their development as advanced therapeutics is hindered by several major challenges. These include low stability of saRNA, degradation and opsonization in the bloodstream, and unintended off-target effects. Utilizing nanoparticle-mediated delivery systems offers a promising solution to these issues.

Liposomes are spherical vesicles composed of one or more phospholipid bilayers and have been widely employed as drug delivery systems due to their excellent biocompatibility, ability to encapsulate both hydrophilic and hydrophobic drugs, and potential for reducing systemic toxicity [13, 14]. Clinically, liposomal formulations have been used for the delivery of chemotherapeutic agents, antibiotics, and vaccines, demonstrating improved pharmacokinetics and therapeutic indices [15]. However, conventional liposomes face several challenges, including limited targeting ability, rapid clearance by the mononuclear phagocyte system, and potential off-target effects [16]. To enhance their specificity and therapeutic efficacy, targeting ligands such as peptides can be incorporated into liposomes, improving their ability to home in on diseased tissues [17, 18].

In recent years, exosomes have emerged as a promising alternative or complementary delivery platform to liposomes. Exosomes are small extracellular vesicles with diameters ranging from 40 to 100 nm, encased in a bilayer lipid membrane [19]. As natural carriers, they offer several advantages including low immunogenicity, high stability in the bloodstream, and efficient drug delivery directly to target cells [20–22]. Exosomes facilitate intercellular communication by transporting various substances and information between cells. By incorporating exogenous drugs such as small-molecule compounds, transmembrane proteins, and nucleic acids, exosomes can effectively alter the functional state of recipient cells. However, challenges related to their specificity and potential off-target effects must be addressed to fully realize their potential in clinical practice. Engineered exosomes are exosomes that have been modified with surface decorations and internal therapeutic molecules. Through these modifications, engineered exosomes can efficiently and precisely deliver antitumor drugs to tumor sites, minimizing treatment-related adverse effects [23, 24].

Metal–organic frameworks (MOFs) are a class of hybrid materials constructed from metal cations and organic bridging ligands. Among them, zinc-based zeolitic imidazolate framework-8 (ZIF-8) has emerged as a promising carrier for nucleic acid delivery due to its high loading capacity, tunable porosity, and favorable biocompatibility [25]. ZIF-8 has been widely reported to efficiently encapsulate RNA molecules through coordination interactions, thereby protecting them from premature degradation in physiological environments [26]. Moreover, ZIF-8 exhibits intrinsic pH-responsive degradability, which is critical for effective intracellular delivery of therapeutic RNAs [27]. Recent studies have demonstrated that ZIF-8-based nanocarriers can safely and effectively deliver siRNA or miRNA in vitro and in vivo with minimal systemic toxicity when appropriately engineered, supporting its suitability as a saRNA delivery platform [28–30].

**Scheme 1:**
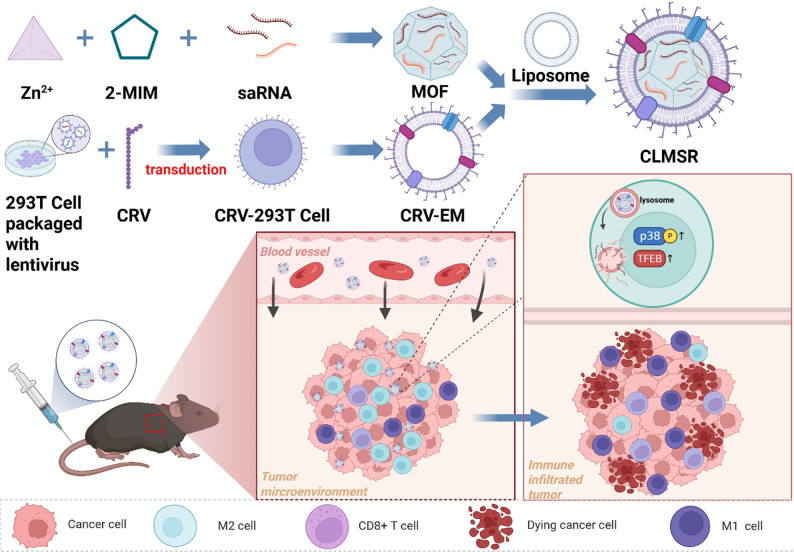
The construction of CLMSR and the induction of TAM transition from the M2 to M1 phenotype. CRV, TAM targeting peptide; CLMSR, CRV-lipo/(MOF/saRNA)

In this study, we developed an innovative antitumor strategy using saRNA encapsulated in engineered nanoparticles to reprogram tumor-associated macrophages (TAMs) toward the M1 phenotype. To specifically target TAMs, we engineered exosomes to overexpress the macrophage-targeting peptide CRV (sequence: CRVLRSGSC) [31] by incorporating it into Lamp2b-expressing plasmids, which were packaged with lentivirus and transfected into HEK 293T cells. The saRNA-loaded ZIF-8 core was then coated with the CRV-expressing exosome membrane and liposomes, forming the multi-layered nanoparticle CLMSR. This design ensures efficient, targeted delivery of saRNA, effective TAM reprogramming, and enhanced antitumor immune responses Scheme 1.

## Materials and methods

### Materials

2-methylimidazole (99%) and zinc nitrate hexahydrate (Zn(NO3)2·6H2O, 98%) were procured from Sigma-Aldrich (USA), while TFEB and p38 were supplied by Genebiogist (Shanghai, China). Dulbecco’s Modified Eagle’s medium (DMEM), Minimum Essential Medium (MEM), serum-reduced medium (Opti-MEM), PBS, penicillin, and streptomycin were acquired from Gibco BRL/Life Technologies (Grand Island, NY, USA). Fetal bovine serum (FBS) was sourced from ExCell Bio (China).

### Cell lines and animals

Raw 264.7 cells were sourced from the National Infrastructure of Cell Line Resource (Beijing, China), whereas Mouse Lewis lung carcinoma cells (LLC) and HEK 293T cells were obtained from ATCC (American Type Culture Collection, USA). Raw 264.7 cells and LLC cells were cultured in DMEM medium, while HEK 293T cells were cultured in MEM medium. The cells were maintained at 37 ℃ in a CO_2_ incubator with the medium supplemented with 10% fetal bovine serum, 100 µg/mL of streptomycin, and 100 U/mL of penicillin.

All the animal experiments were approved by the Institutional Animal Care and Use Committee of Guangzhou Medical University (GY2023-647). The C57BL/6 mice aged 5–6 weeks were purchased from Guangdong Vital River Laboratory Animal Technology Co., Ltd.

### Establishment of CRV-expressing HEK 293T cells

A peptide (amino acid sequence, CRVLRSGSC, abbreviated as CRV) that selectively bound to TAMs was previously described [32, 33]. Following a previously established protocol [34], 10 µg CRV-Lamp2 fusion plasmid, 5 µg pVSV-G, 5 µg pRSV-REV, and 5 µg pMDL g/p RRE were mixed with Opti-MEM medium. The plasmid solution was added to diluted Lipofectamine 3000 and incubated for 20 min, followed by addition of the mixture to the culture medium of HEK 293T cells. After 6 h of incubation, the medium was replaced with fresh DMEM medium containing 10% FBS and cultured for 48 h. The transfected cells were then passaged and cultured in DMEM medium containing 5 µg/mL puromycin until colony formation. Subsequently, the colonies were digested and cultured in DMEM to obtain HEK 293T cells expressing CRV.

### Agarose gel electrophoresis migration transfer test

The electrophoretic mobility shift assay was employed to assess the formation and encapsulation efficiency of MOF/saRNA. Various ratios of MOF/saRNA (2-methylimidazole/saRNA = 10:1, 25:1, 50:1, 75:1, 100:1, 150:1) were loaded onto agarose gel electrophoresis and separated at 80 V for 40 min. Gel images were captured using the Amersham Imager 600 system (GE Healthcare Life Sciences, USA).

### Isolation and identification of exosomes

To isolate the CRV-Exosomes, the CRV-expressed HEK 293T cells were cultured in 150 mm dishes for 48 h. The medium was initially centrifuged at 3000 g for 5 min to eliminate dead cells and cell debris. Subsequently, the supernatant was further centrifuged at 10,000 g for 15 min at 4 °C followed 100,000 g for 70 min at 4 °C to acquire exosomes. The exosome membrane was dissolved using reagent A from the Cytosol Protein Extraction Kit (Beyotime Biotechnology, Shanghai, China) in PBS and stored at -80 °C for subsequent experiments. The protein expression of HSP70 (1:1000, 70KDa, Abcam), Alix (1:1000, 109KDa, Abcam), and FLAG (1:1000, 55KDa, Abcam) in the exosome membrane from HEK 293T cells expressing CRV was analyzed using Western blot analysis.

### Synthesis of MOF/saRNA (MSR) and lipo/(MOF/saRNA) (LMSR), CRV-lipo/(MOF/saRNA) (CLMSR)

2-methylimidazole (200 mg/mL) and p38, TFEB (1 mg/mL) were dissolved separately in RNase-free water. The solutions were stirred for 10 min, followed by the addition of Zn (NO3)2·6H2O (10 mg/mL) and continued stirring. The mixture was stirred in the dark at room temperature for 10 min, then subjected to centrifugation (12,000 rpm, 15 min) to separate MOF/saRNA from the dispersion. To screen for the appropriate ratio of p38 to TFEB, the mass ratio (w/w) of p38 to TFEB was set as 1:1, 1:2, and 2:1, respectively. By designing the mass ratio (w/w) of MOF (2-methylimidazole: Zn (NO_3_)_2_·6H_2_O = 2:1) to saRNA as 10:1, 25:1, 50:1, 75:1, 100:1, and 150:1, the suitable ratio of MOF to saRNA could be screened. SM-102, cholesterol, distearoylphosphatidylcholine (DSPC), and DMG-PEG 2000 were mixed at a ratio of 1:4:2:9 (10 mg/mL) in chloroform, and the organic solvent was evaporated under reduced pressure to form a lipid membrane. The solution containing MOF/saRNA was then added to obtain lipo/(MOF/saRNA) (Lipo: saRNA = 20:1). CRV and lipo were combined in a weight ratio of 1:5, subjected to 5 min of sonication, and then extruded to obtain CRV-lipo/(MOF/saRNA).

CLMSR-NC nanoparticles were prepared following the same procedure, encapsulating a non-coding (scrambled) saRNA sequence with no known biological activity, serving as a negative control.

### Characterization of nanoparticles

The Nano ZS instrument (Malvern, UK) was employed to measure the polydispersity index (PDI), particle size, and zeta potential of MOF/saRNA, lipo/(MOF/saRNA), and CRV-lipo/(MOF/saRNA). The morphology of the three samples was examined using a transmission electron microscope (JEM2000FX, Hitachi). The encapsulation efficiency of saRNA was assessed by detecting unbound saRNA in the supernatant. Subsequently, the unbound saRNA content in the supernatant was analyzed using agarose gel electrophoresis.

### Cell viability assay

The cellular toxicity of MOF/saRNA, lipo/(MOF/saRNA), and CRV-lipo/(MOF/saRNA) was assessed using live/dead cell staining and the CCK-8 assay. LLC cells were seeded in a 96-well plate at a density of 3 × 10^3 cells per well and cultured for 24 h. Subsequently, the conditioned medium from M2 macrophages treated with PBS, CRV-lipo/(MOF/saRNA-NC), Free saRNA, MOF/saRNA, and lipo/(MOF/saRNA), CRV-lipo/(MOF/saRNA) was applied and incubated for 48 h. The cells were then stained for live/dead cells using a detection kit (Life Technology, USA).

Afterward, cell viability was evaluated using the CCK-8 assay kit (Beyotime Biotechnology, China) in accordance with the manufacturer’s instructions. Subsequently, the ratio of cell viability was calculated as A _treated_/A _control_×100%.

### Reverse transcription-quantitative polymerase chain reaction (RT-qPCR) assay

First, total RNA was extracted using the Trizol reagent, and 500 ng of total RNA was used for cDNA synthesis with the Evo M-MLV RT Kit, following the manufacturer’s protocol. Subsequently, 1 µL of cDNA and SYBR Green were used for qPCR detection. The primers employed for RT-qPCR are listed in the Table S1. Differences in target expression were quantitatively analyzed using GAPDH as the internal reference gene.

### Western blotting (WB) assay

An equal amount of total protein was loaded onto 10% SDS-PAGE gels, transferred to PVDF membranes (300 mA for 2 h), and then probed with primary antibodies. The primary antibodies used were anti-Arg-1 (1:1000, 35 kDa, Abcam), anti-CD206 (1:1000, 175 kDa, Abcam), anti-CD80 (1:1000, 60 kDa, Abcam), anti-iNOS (1:1000, 131 kDa, Abcam), and anti-β-tubulin (1:1000, 50 kDa, Affinity Biosciences). The protein bands of interest were captured after the secondary antibodies linked with peroxidase were bound to the primary antibodies.

### Extracellular cell uptake

RAW264.7 cells were seeded in confocal culture dishes at a density of 1 × 10^5 cells per well and cultured in Opti-MEM containing 50 ng/mL for two days to induce the M2 phenotype. Afterward, the RAW264.7 cells induced as M2 macrophage were exposed to CRV-lipo/(MOF/saRNA) nanoparticles at different concentrations (Cy5-saRNA: 0.25, 0.5, 1, 2 µg/mL; Cy5-saRNA: Cy3-saRNA = 2:1) in complete DMEM medium for 12 h. Following incubation, the cells were stained with DAPI and Actin-Tracker Green. To investigate the temporal dynamics of nanoparticle uptake by cells, different incubation times (1, 3, 6, and 9 h) were designed, and the aforementioned method was utilized. Confocal laser scanning microscopy (CLSM, LSM880, Zeiss, Germany) was utilized to qualitatively demonstrate the cellular uptake of the nanoparticles.

Flow cytometry analysis (FACS) was employed for the quantitative evaluation of cellular uptake. RAW264.7 cells were treated with different concentrations of CRV-lipo/(MOF/saRNA) nanoparticles (Cy5-saRNA at 0.25, 0.5, 1, and 2 µg/mL, with a Cy5-saRNA: Cy3-saRNA ratio of 2:1). Similarly, the time-dependent uptake kinetics were studied using the same method at 1, 3, 6, and 9 h. Cells were collected and suspended in PBS for analysis using flow cytometry (FACS, CytoFLEX, Beckman Coulter, USA).

To assess the uptake efficiency of CRV-lipo/(MOF/saRNA), the same method was employed to co-incubate the M2-type RAW264.7 cells with the same concentration of free saRNA, MOF/saRNA, lipo/(MOF/saRNA), and CRV-lipo/(MOF/saRNA) for 6 h. The cellular fluorescence of Cy3 and Cy5 was analyzed by CLSM and FACS.

### Lysosome escape assay

To assess the intracellular release of saRNA, lysosome escape assays were conducted. RAW264.7 cells induced to the M2 phenotype were treated with CRV-lipo/(MOF/saRNA) (Cy5-saRNA at a concentration of 1 µg/mL, with a Cy5-saRNA: Cy3-saRNA ratio of 2:1) for 3 and 9 h. Subsequently, the cells were stained with Lyso-Tracker Blue DND-22 (Thermo Fisher, USA), and the distribution of nanoparticles at different time points was detected and visualized by confocal laser scanning microscopy (CLSM).

### Wound healing and transwell invasion assays

Wound healing and Transwell invasion assays were conducted following previously published methods [35]. These assays were used to evaluate the effects of various treatments, including CLMSR-NC, free saRNA, MSR, LMSR, or CLMSR, on cell invasion and migration.

### The evaluation of CLMSR in 3D tumor spheroids

A 2% agarose solution was prepared by dissolving agarose in DMEM. To coat the bottom of each well in a 96-well plate, 60 µL of this agarose solution was used. LLC cells (4 × 10^3 cells/well) were seeded into the agarose-coated plate and cultured for 5 days to form approximately 500 μm 3D tumor spheroids.

After the formation of these tumor spheroids, they were transferred to agarose-coated confocal dishes and treated with CLMSR-NC, free saRNA, MSR, LMSR, or CLMSR for 24 h. The treated cellular spheroids in the confocal dishes were stained using the Live/Dead Cell Viability Assay Kit and imaged using the Z-stack function of CLSM.

### In vivo tracking study

To investigate the targeting effect of nanoparticles in vivo, we prepared Cy5-labeled free saRNA (20 µg/mouse), MOF/saRNA (containing 20 µg of saRNA per mouse), lipo/(MOF/saRNA) (containing 20 µg of saRNA per mouse), and CRV-lipo/(MOF/saRNA) (containing 20 µg of saRNA per mouse) for tracking studies. Male C57BL/6 mice were implanted with 2 × 10^6 LLC cells in the right region. Once the tumor reached approximately 200 mm^3 in size, the prepared nanoparticles were intravenously injected through the tail vein of the LLC tumor-bearing C57BL/6 mice.

At 1–48 h post-injection, in vivo fluorescence imaging was performed using an IVIS Lumina imaging system. Cy5-labeled saRNA was excited at 640 nm and fluorescence emission was collected at 680 nm. Whole blood samples were collected at designated time points to measure Cy5 fluorescence intensity. The mice were euthanized under tribromoethanol anesthesia, followed by imaging of the heart, liver, spleen, lungs, kidneys, and tumor.

### Animal experiments

Male C57BL/6 mice weighing approximately 18–22 g were randomly divided into six groups (*n* = 4) for subcutaneous transplantation of LLC tumors. Each group of mice was injected via tail vein with saline, CRV-lipo/(MOF/saRNA-NC), Free-saRNA (20 µg/mouse), MSR (20 µg/mouse), LMSR (20 µg/mouse), and CLMSR (20 µg/mouse), respectively. Body weight and tumor volume were recorded throughout the experiment. After the experiment, the mice were euthanized immediately, and major organs (heart, lungs, liver, spleen, kidneys) and tumors were excised. The collected tissues and tumors underwent hematoxylin & eosin (H&E) staining, terminal deoxynucleotidyltransferase-mediated dUTP-biotin nick end labeling (TUNEL)analysis, and immunofluorescence staining.

### Immunofluorescence staining

Tumor tissue sections were incubated with anti-iNOS and anti-Arg1 antibodies, followed by pairing with goat anti-rabbit IgG H&L (FITC) or goat anti-rabbit IgG-H&L (TRITC) for secondary antibody reaction. The nucleus was stained with DAPI. Confocal microscopy (Zeiss, LSM880, Germany) was used to observe the target protein.

### Statistical analysis

Data were analyzed using GraphPad Prism 8.0 software. All data are presented as mean ± standard deviation (SD). The unpaired two-tailed Student’s t-test was used for comparison, and one-way ANOVA or two-factor ANOVA was performed for comparisons between two or more groups. The log-rank test was used to determine the statistical significance of the survival curve. A significance level of *P* < 0.05 was considered significant.

## Results

### The effect of p38 and TFEB on macrophage phenotype transition

To ensure the efficient function of saRNA, 6 sequences of p38 and TFEB saRNA were designed, respectively. The sequences with the highest activation efficiency (p38-5 and TFEB-1 saRNA) were selected for further experiment (Figure S1). To explore the effects of different ratios of p38 and TFEB on the phenotype transition of M2 macrophages, the commercial Lipofectamin3000 was used for transfection. RT-qPCR analysis revealed that when the ratio of p38 to TFEB was 2:1, the mRNA expression levels of M1 macrophage markers (*Nos2* and *Cd80*) were significantly upregulated, while those of M2 markers (*Mrc1* and *Arg1*) were notably downregulated (Figure S2A). Consistently, western blotting results showed a corresponding increase in the protein expression of CD80 and iNOS, along with a marked decrease in Arg1 and CD206 protein levels under the same conditions (Figure S2B). Consequently, the ratio of p38-5 to TFEB-1 2:1 was selected for subsequent experiments. When the ratio of p38 to TFEB was 2:1, we tested different concentrations of these compounds. We found that none of the tested combinations exhibited toxicity towards M2 macrophages (Figure S3A). We also detected the cell viability of blank MOF (without saRNA) at the same concentrations applied in our formulations. In addition, macrophages were treated with MSR, LMSR, CLMSR, and CLMSR-NC. The results showed that none of these treatments caused detectable cytotoxicity to macrophages, indicating good biocompatibility of the MOF core as well as the assembled nanoplatforms (Figure S3B).

MOF was used to carry saRNAs. The encapsulation efficiency of saRNA by MOF was determined through agarose gel electrophoresis of the unbound saRNA in the supernatant. Results revealed an encapsulation efficiency of 84.4% at a 2-methylimidazole to saRNA ratio of 100:1, and 99.2% at a ratio of 150:1 (Figure S4). Considering these factors, MOF/saRNA with a 2-methylimidazole to saRNA mass ratio of 100:1 was selected for subsequent experiments.

### Characterization of nanoparticles

**Fig. 1:**
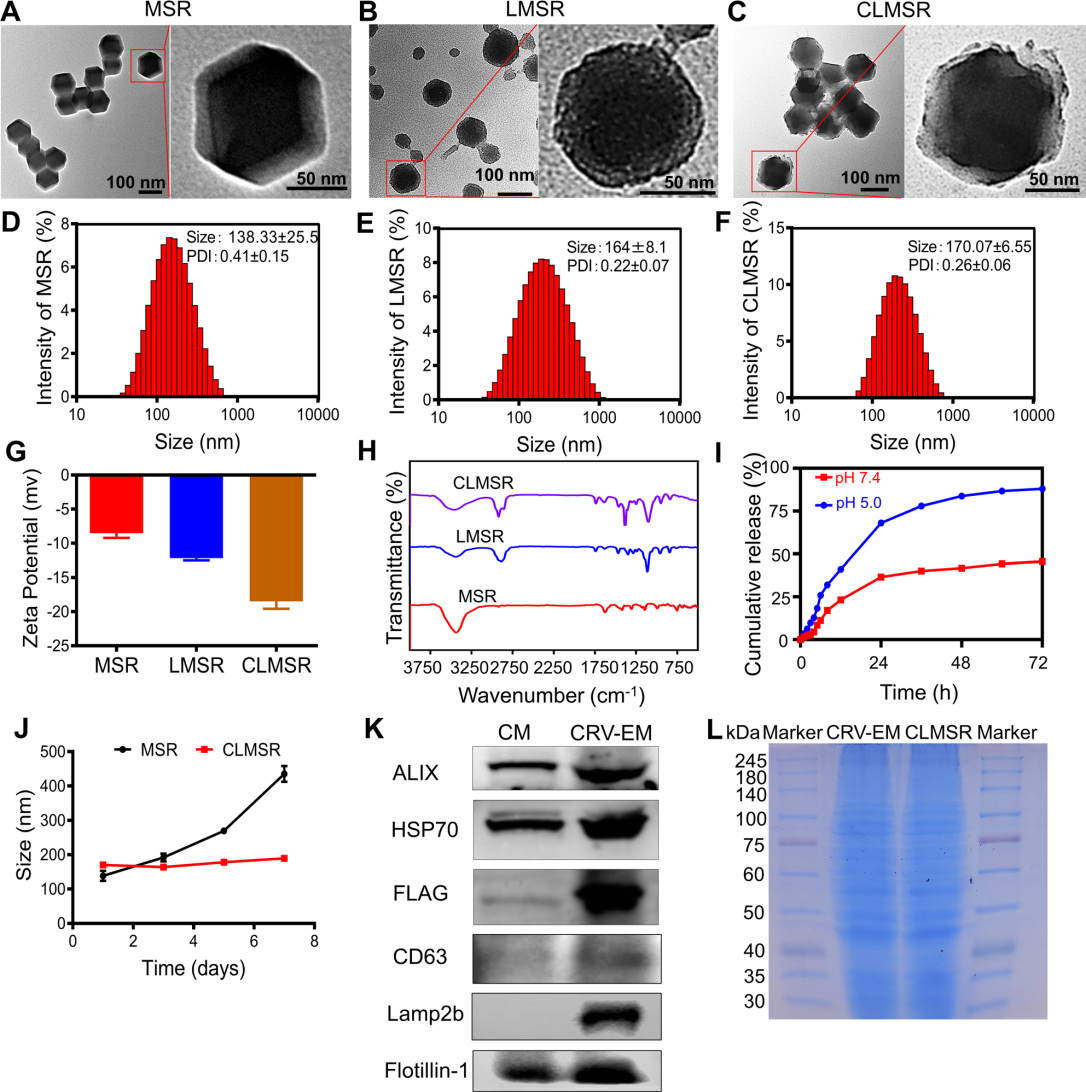
Characterization of saRNA-based nanoparticles. (**A-C**) TEM images of MSR, LMSR and CLMSR. (**D-F**) DLS analysis of MSR, LMSR and CLMSR. (**G**) DLS analysis of MSR, LMSR and CLMSR. (**H**) FTIR spectra of MSR, LMSR and CLMSR. (**I**) The cumulative release profile of saRNA from CLMSR in neutral (pH = 7.4) and acidic environment (pH = 5.0). (**J**) The stability of MSR and CLMSR in 7 days. (**K** and **L**) Characterizations of retained proteins on the CRV-EM and CLMSR

Transmission electron microscopy (TEM) was employed to examine the nanoparticles. TEM images revealed a diamond-shaped polyhedral three-dimensional structure with well-defined edges, confirming the successful synthesis of MOF/saRNA (MSR) (Fig. 1A). In contrast, the morphology of lipo/(MOF/saRNA) (LMSR) and CRV-lipo/(MOF/saRNA) (CLMSR) appeared rough surface, indicating successful loading and surface modification of liposomes and lipid/exosome membranes (Fig. 1B and C). MSR, LMSR and CLMSR all displayed uniformly dispersed nanoparticles with average diameters of 138.33 ± 25.51 nm, 164.33 ± 8.1 nm, and 170.07 ± 6.55 nm, respectively (Fig. 1D-F). The zeta potential of MSR was − 8.54 mV, while for LMSR, it was − 12.2 mV. Upon coating with mixed membranes, the zeta potential of the nanoparticles decreased to -18.5 mV (Fig. 1G), indicating enhanced stability compared to MSR alone (Fig. 1G). As shown in Fig. 1H, Fourier transform infrared spectroscopy (FTIR) spectra of MSR, LMSR and CLMSR exhibit characteristic peaks at 3438 cm^− 1^, 1142 cm^− 1^, and 1630 cm^− 1^, corresponding to the N-H, C-N, and C = N vibrations of 2-methylimidazole, confirming its presence. A peak at 421 cm^− 1^, attributed to the Zn-N bond in MOF, indicates successful synthesis of MOF in all formulations. Additionally, absorption peaks in the 1100–1300 cm^− 1^ range suggest the successful loading of saRNA onto MOF. For LMSR and CLMSR, new peaks at 1737 cm^− 1^ and 2887 cm^− 1^ are observed, indicating the presence of liposomes and membranes. CLMSR also shows an enhanced absorption in the 1100–1300 cm^− 1^ region and a shift of the carboxylic acid peak to lower wavenumbers, forming a broad peak around 3200 –2500 cm^− 1^.

We observed that CLMSR exhibited a pH-responsive release of saRNA, with approximately 90% released within 72 h at pH 5.0 (Fig. 1I). In contrast, at pH 7.4, the cumulative release of saRNA was around 50% over the same period (Fig. 1I). The particle size of CLMSR was continuously monitored over a period of 7 days, and no significant changes were observed. This consistent particle size indicated that the mixed membrane coating provided substantial stability to the nanoparticles. In contrast to MSR, the CLMSR with the mixed membrane encapsulation exhibited significantly enhanced stability (Fig. 1J).

Western blot analysis confirmed the successful construction of CRV-expressed exosome membranes (CRV-EM). Lamp2b and the FLAG-tagged Lamp2b–CRV fusion protein demonstrated successful surface display of the CRV targeting peptide. Exosomal markers ALIX, HSP70, and CD63 were also detected, confirming the exosomal origin of the membrane. Flotillin-1, expressed in both cellular and exosomal membranes, was used as a reference protein to ensure consistent membrane protein loading (Fig. 1K). After the saRNA was complexed by MOF, the lipid/exosome membranes were used to coat the MSR. To verify the successful loading of CRV exosome membranes in LMSR, sodium dodecyl sulfate-polyacrylamide gel electrophoresis (SDS-PAGE) was utilized. The SDS-PAGE results demonstrated that CLMSR retained most of the proteins from CRV-EM (Fig. 1L), confirming the successful integration of CRV exosome membranes into the mixed membrane on the surface of CLMSR.

### Cellular uptake assessment

The cellular uptake of CLMSR was assessed at various time points and concentrations to elucidate its uptake kinetics and dose-dependence in M2 macrophages. Confocal laser scanning microscopy (CLSM) and flow cytometry FACS analysis revealed that the cellular uptake of CLMSR increased with both time and dosage. At a concentration of 1 µg/mL, the cellular uptake of CLMSR was approximately 87.26%, whereas after 12 h of incubation at 2 µg/mL, the cellular uptake reached around 90.71%. Further increases in CLMSR concentration did not significantly enhance the cellular uptake (S5A). Additionally, after the incubation for 6 h of at a concentration of 1 µg/mL, the cellular uptake reached 88.65%, with longer incubation time showing no significant improvement (S5B). Consequently, subsequent experiments were conducted under optimal conditions, incubating CLMSR at a concentration of 1 µg/mL for 6 h.

**Fig. 2:**
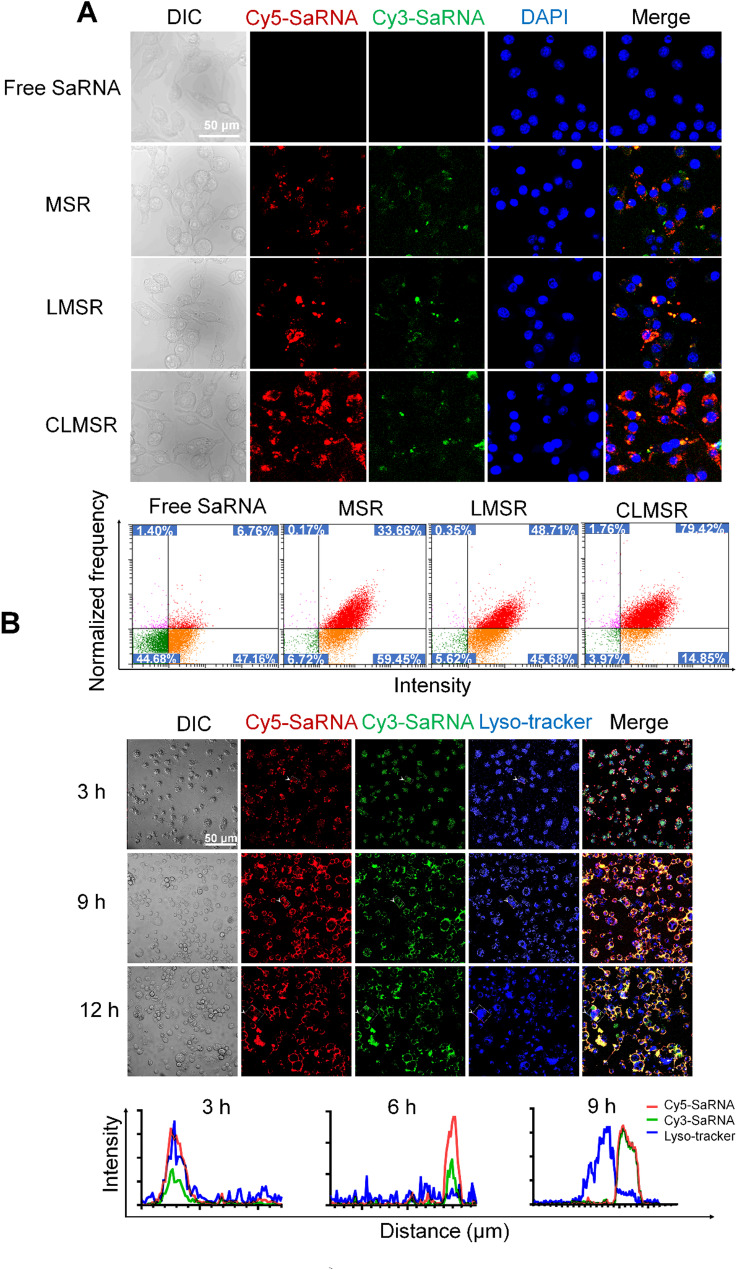
Cellular uptake of CLMSR in induced M2 macrophages observed by CLSM. (**A**) Comparison of cellular uptake across different nanoparticles. (**B**) Lysosome escape induced by CLMSR

We also analyzed the cellular uptake of other formulations. Both CLSM and FACS analysis indicated that the cellular uptake was only 6.76% when only free saRNA was present. However, when saRNA was loaded onto MOF, the uptake efficiency increased to 33.66%. For LMSR, the uptake efficiency was 48.71%. In comparison, the cellular uptake of CLMSR significantly increased to 79.42%, indicating a more effective cellular uptake of CLMSR (Fig. 2A).

To evaluate the lysosomal escape capability of CLMSR nanoparticles, lysosomes were labeled with the fluorescent dye LysoTracker, while P38 and TFEB were respectively tagged with red fluorescent dyes Cy5 and Cy3. Following the synthesis of CLMSR, cells were incubated with the nanoparticles for 3, 9, and 12 h. The intracellular localization of the nanoparticles in relation to lysosomes was then examined using confocal laser scanning microscopy (CLSM). CLSM analysis revealed that, after 3 h of incubation, a substantial amount of CLMSR nanoparticles colocalized with lysosomes, indicating that they were largely sequestered within these organelles (Fig. 2B). However, after 9 h, a significant proportion of Cy5-labeled SaRNA (P38) and Cy3-labeled SaRNA (TFEB) was observed to be separated from the lysosomes, suggesting effective lysosomal escape of the nanoparticles (Fig. 2B). By 12 h, nearly all fluorescent signals from Cy5 and Cy3 were detected outside lysosomal compartments, confirming that CLMSR nanoparticles had completely escaped from lysosomes (Fig. 2B). These results demonstrate that CLMSR nanoparticles exhibit time-dependent lysosomal escape, which is critical for the efficient cytoplasmic delivery of functional RNA cargos.

#### The effect of CLMSR on macrophage phenotype transition

**Fig. 3:**
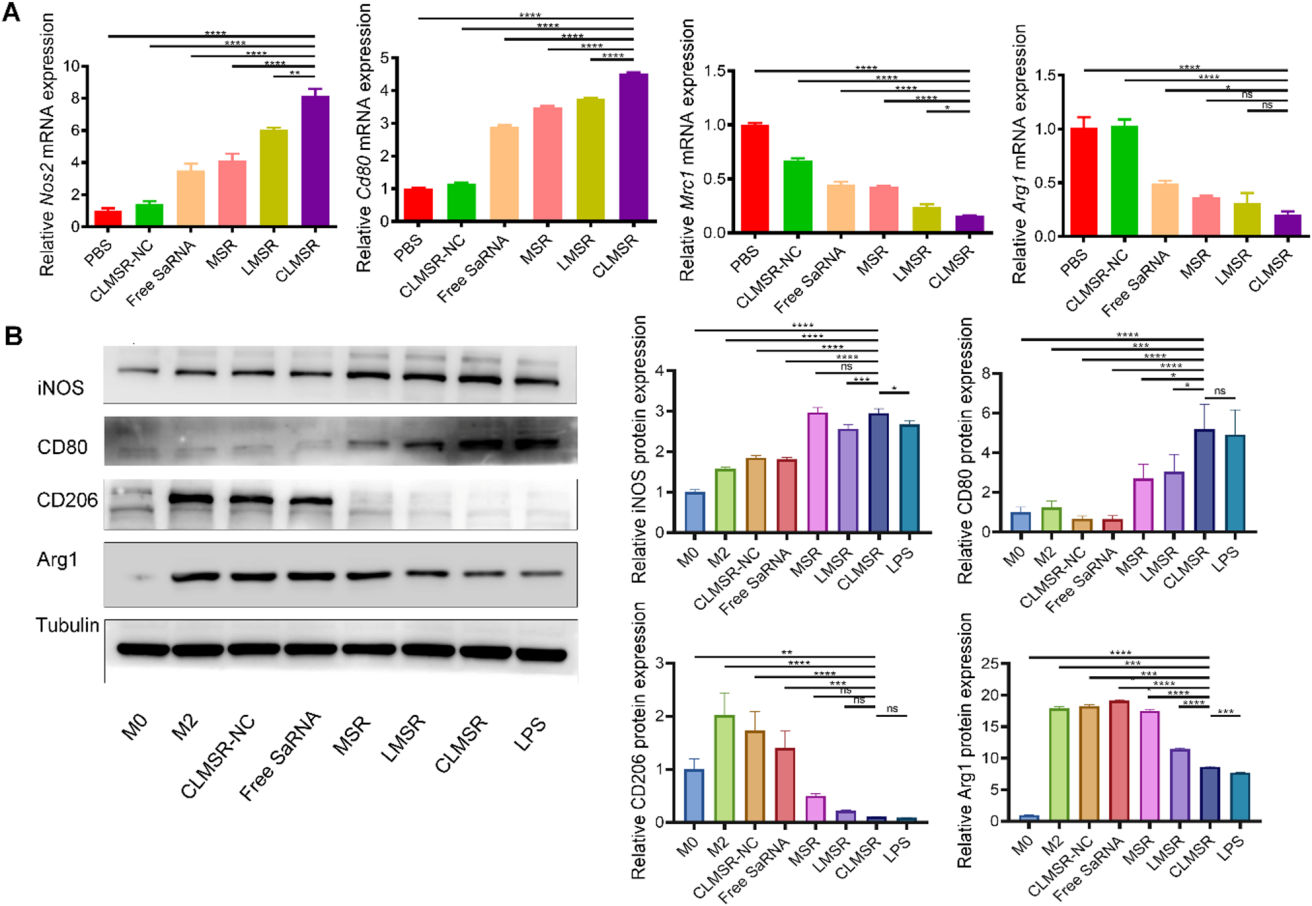
The effect of CLMSR on macrophage phenotype transition. (**A**) The mRNA expression levels of *Nos2*, *Cd80*, *Mrc1* and *Arg1* were measured by RT-qPCR. (**B**) Western blotting and densitometric quantification of iNOS2, CD80, CD206, and Arg1. All data are presented as the mean ± SD; ns, no significance; ^*^P < 0.05, ^**^P < 0.01, ^***^P < 0.001, and ^****^P < 0.0001 vs. treatment with CLMSR group

The impact of various formulations on macrophage phenotype transition was assessed through RT-qPCR and western blot (WB) assays. RT-qPCR results indicated that the treatment with CLMSR significantly increased the expression of *Nos2* and *Cd80* while decreasing the expression of *Mrc1* and Arg1 compared to other groups in the induced M2 macrophages (Fig. 3A), implying the effective induction of macrophages to the M1 phenotype. WB analysis further supported these findings, demonstrating that CLMSR elevated the levels of M1 markers (CD80 and iNOS) and reduced the levels of M2 markers (Arg1 and CD206) (Fig. 3B). This further confirmed that CLMSR successfully induced the transition of macrophages to the M1 phenotype.

Bright-field microscopy showed that Raw264.7 M2 macrophages exhibited an elongated, spindle-like morphology with smooth edges and few pseudopodia, reflecting weak adhesion. In contrast, CLMSR-treated macrophages adopted a flattened, irregular M1-like morphology with abundant pseudopodia and enhanced adhesion (Figure S6A). Consistently, flow cytometry revealed a significant increase in CD80⁺ (M1) macrophages and a marked decrease in CD206⁺ (M2) macrophages relative to controls, quantitatively confirming that CLMSR reprograms macrophages from an M2- to an M1-dominant phenotype (Figure S6B).

### *In vitro* antitumor activity

**Fig. 4:**
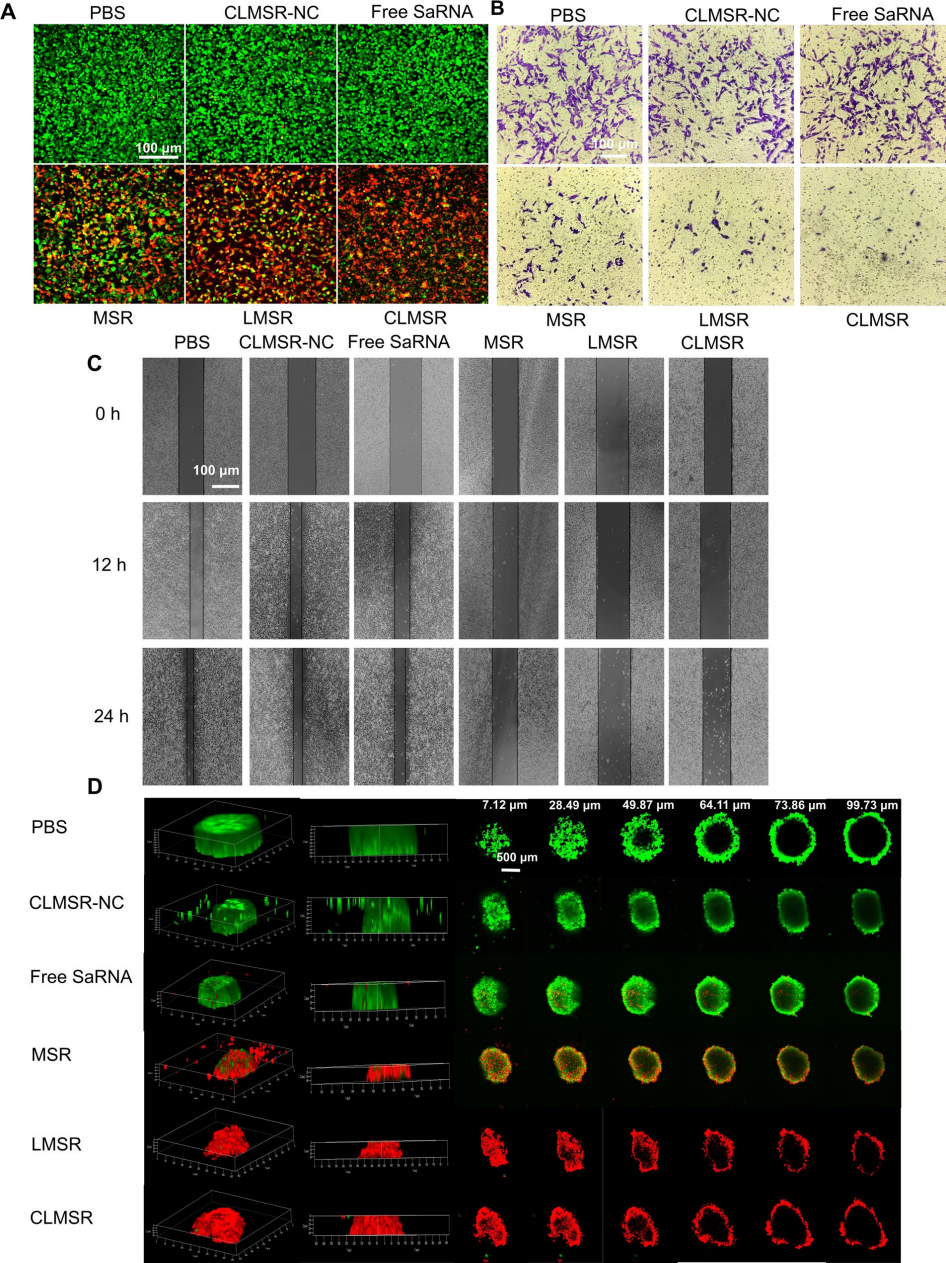
In vitro antitumor activity of CLMSR. (**A**) Live/dead staining, (**B**) cell invasion, (**C**) cell migration and (**D**) 3D tumor spheroids were analyzed after treatment with PBS, CLMSR-NC, free saRNA, MSR, LMSR and CLMSR, respectively

We evaluated the cancer cell inhibition induced by the induced M1 macrophages. The spent medium from the CLMSR induced M1 macrophages was collected for the further experiments. The inhibitory effect on cancer cells was evaluated in vitro using LLC cells and three-dimensional (3D) spheroids. Live/Dead assay and CCK-8 assay revealed that the conditioned medium from M1 macrophages induced by CLMSR significantly inhibited the proliferation of cancer cells after 48 h of incubation (Fig. 4A and S7A). The spent medium from the induced M1 macrophages also inhibited the migration and invasion of LLC cells, with the most pronounced effect observed in the treatment group with CLMSR (Fig. 4C and B, S7C and S7B). In addition, the conditioned medium from these induced M1 macrophages suppressed the growth of 3D cultures of LLC cells and induced cell death within these cultures, as evidenced by the Live/Dead assay (Fig. 4D). Moreover, we monitored the size of tumor spheroids in different treatment groups under bright-field microscopy on days D2, D4, D6, D8, and D10. The results showed that spheroids in the CLMSR-treated group were significantly smaller compared to the control groups, demonstrating a pronounced growth inhibition effect (Figure S7D). These results highlight the potent antitumor activity of CLMSR through the reprogramming of TAMs to an M1 phenotype, effectively suppressing tumor growth in a controlled environment.

#### Biodistribution in vivo

**Fig. 5:**
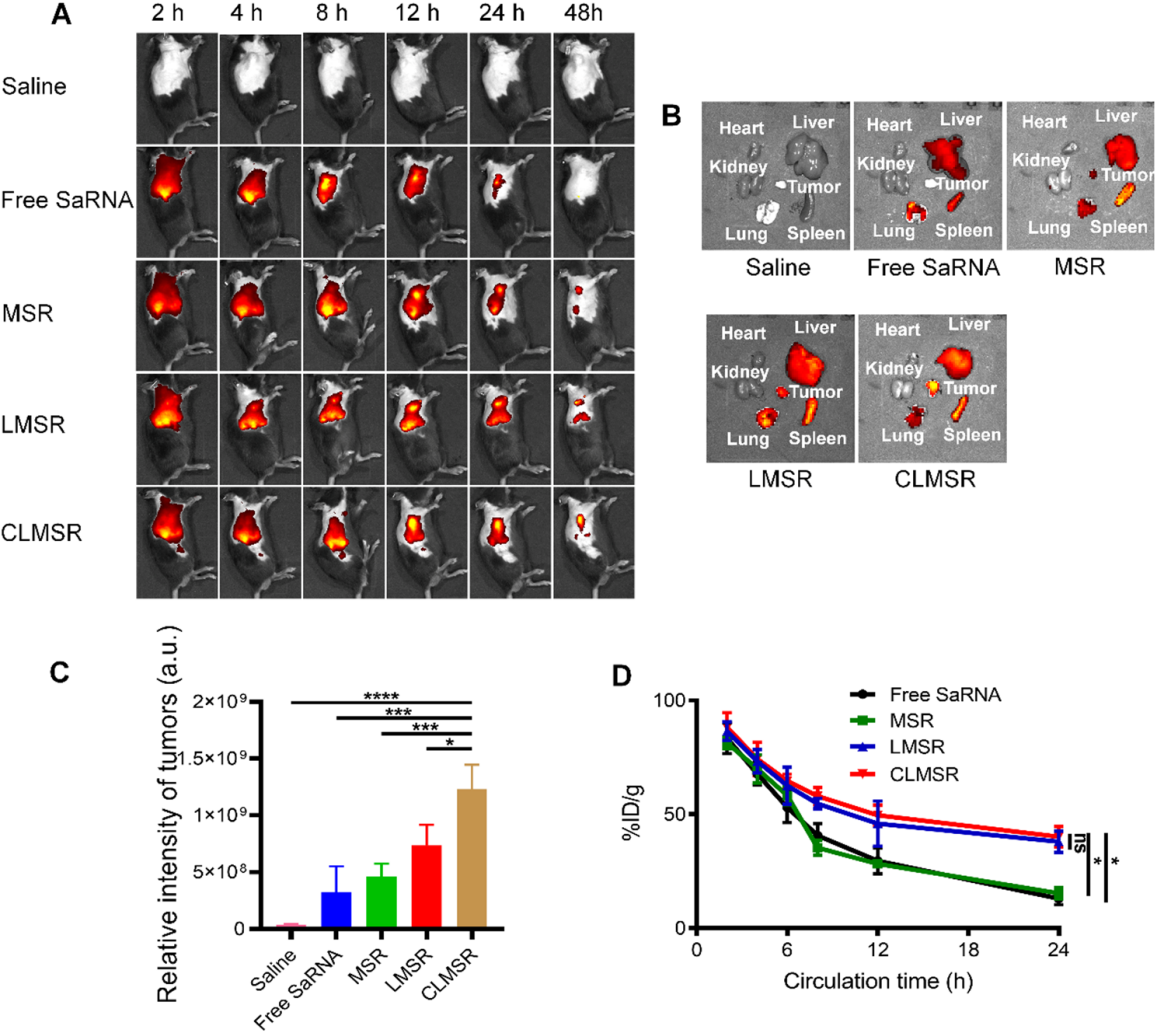
In vivo biodistribution and circulation of various nanoparticles. (**A**) In vivo biodistribution of nanoparticles. (**B**) Ex-vivo images of major organs. (**C**) Quantitative fluorescence analysis in major organs and tumors. (**D**) Circulation lifetime of nanoparticles. All data are presented as the mean ± SD; ns, no significance; ^*^P < 0.05, ^**^P < 0.01, ^***^P < 0.001, and ^****^P < 0.0001 vs. treatment with CLMSR group

To monitor the biodistribution and tumor accumulation of CLMSR-NC, LLC tumor-bearing mice were administered Cy5-labeled saRNA. As depicted in the Fig. 5A, within 2 h post-intravenous injection, CLMSR exhibited widespread distribution throughout the body. Compared to the CLMSR-NC, free saRNA, MSR, or LMSR group, the CLMSR group showed higher signal intensity, whereas no fluorescence signal was detected in the saline-treated group. At 48 h post-injection, the organs and tissues were harvested. At the tumor site, CLMSR exhibited significantly stronger red fluorescence compared to the other groups (Fig. 5B and C), indicating that the mixed membrane containing CRV exosome membranes enhanced the selective targeting of saRNA to tumor sites.

The circulation half-life of CLMSR and other formulations was assessed. Six hour post-administration, the CLMSR group displayed a significant increase in blood circulation lifespan by over 60%, with an approximately 40% retention after 24 h (Fig. 5D). The prolonged blood circulation extended interaction time between the nanodrug system and the lesion site, enhancing targeting efficiency and drug concentration at the tumor site.

The in vivo biodistribution and circulation lifetime demonstrated that CLMSR enabled to accumulated in the tumor sites effectively. On one hand, the macrophage specific peptide CRV may increase the binding to macrophages in the tumors. On the other hand, the biomimetic surface can reduce the non-specific capture by the immune systems, and the extension of circulation lifetime increases the probability to the tumors.

### *In vivo* antitumor effects of CLMSR

**Fig. 6:**
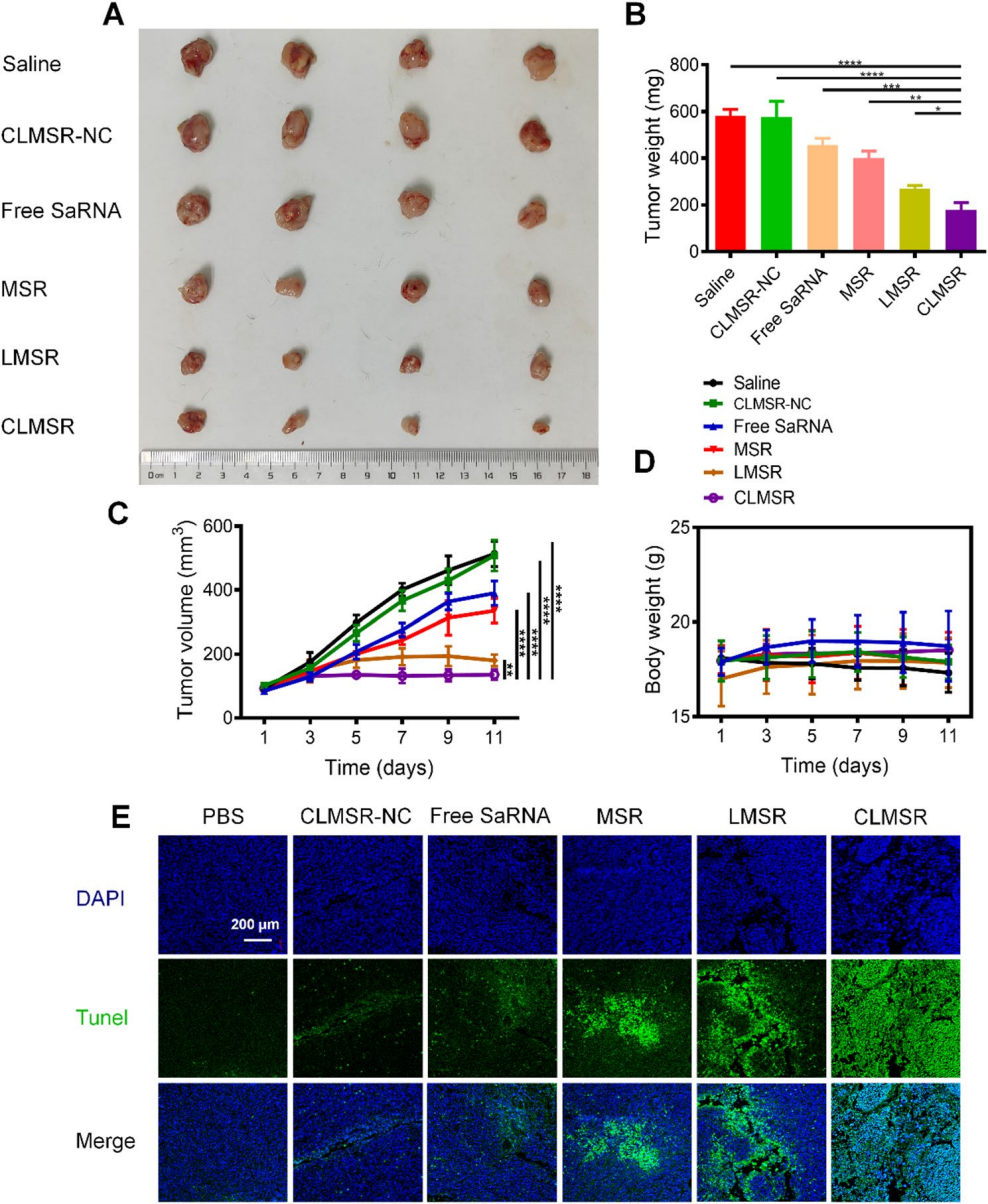
In vivo antitumor effects of CLMSR. (**A**) Imaging of the excised tumors. (**B**) Weighing of the excised tumors. (**C**) Measurement of changes in tumor volumes. (**D**) Monitoring of body weight changes. All data are presented as the mean ± SD; ns, no significance; ^*^P < 0.05, ^**^P < 0.01, ^***^P < 0.001, and ^****^P < 0.0001 vs. treatment with CLMSR group

We evaluated the therapeutic efficacy of CLMSR in LLC tumor-bearing mice. Different formulations including saline, CLMSR-NC, free saRNA, MSR, and LMSR were administered via tail vein injection. Tumor volume and body weight of the mice were regularly monitored throughout the treatment period. As illustrated in Fig. 6A-C, no significant differences in tumor size, weight, and volume were observed in the mice treated with saline or CLMSR-NC. Conversely, the substantial therapeutic effects were observed in the CLMSR treated group. In contrast, the The excised tumors confirmed the therapeutic effects of the different formulations, demonstrating that CLMSR was the most effective in suppressing tumor growth. Throughout the treatment process, we monitored the mice’s body weights and found no significant weight loss (Fig. 6D), indicating that the CLMSR treatment is a safe and effective approach for tumor therapy.

TUNEL staining was performed to assess apoptosis in tumor tissues, revealing a higher apoptotic rate in mice treated with free saRNA, MSR, LMSR, and CLMSR compared to those treated with physiological saline or CLMSR-NC, with the highest rate observed in the CLMSR group (Fig. 6E). H&E staining revealed no signs of major organ tissue damage or structural disarray in any treatment groups, indicating minimal toxicity to the mice. Notably, necrotic areas were evident in the tumor tissues of the saRNA nanoparticle-treated group, whereas CLMSR group exhibited the most extensive necrosis (Figure S8).

**Fig. 7:**
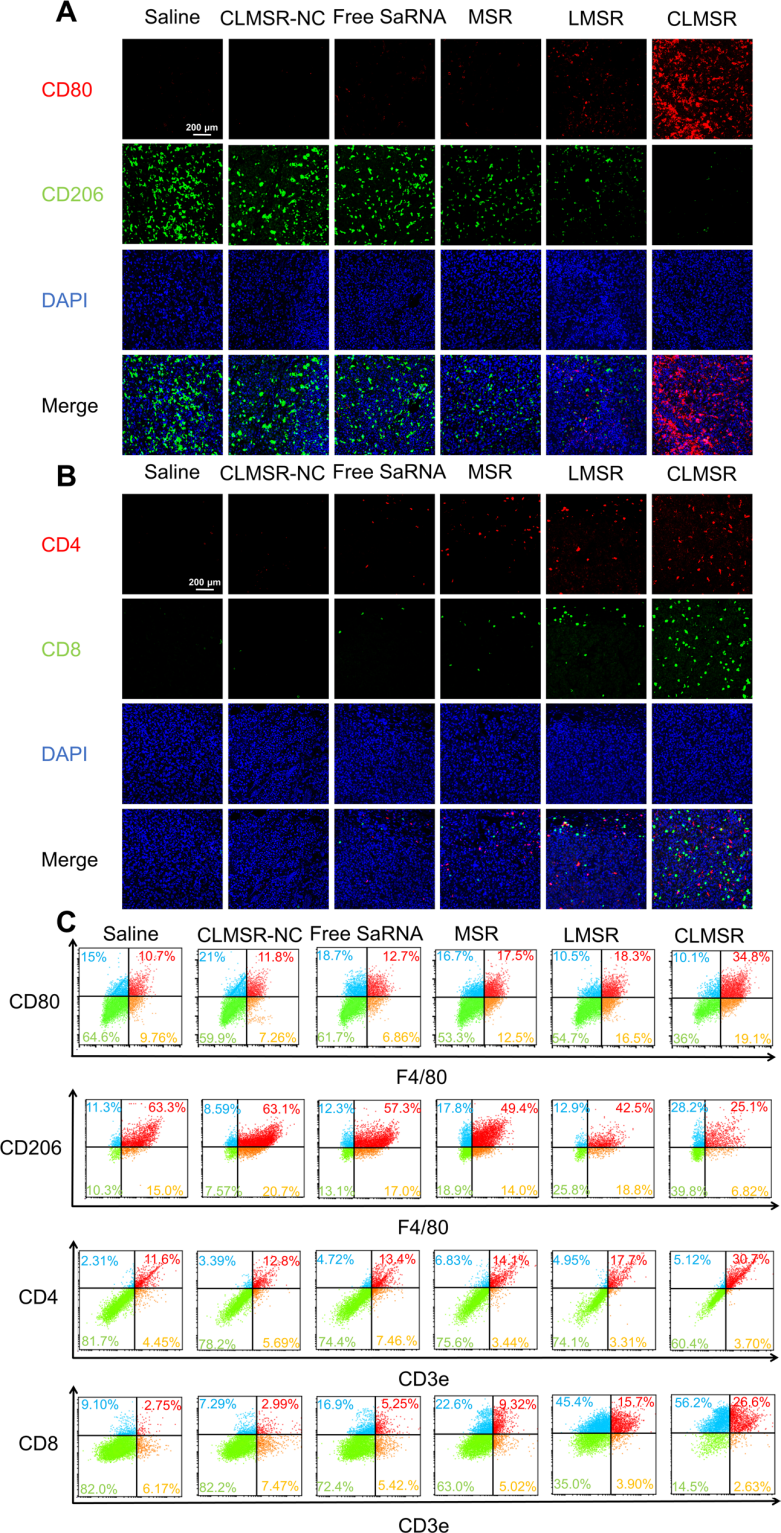
Macrophage polarization profiles and intratumoral T cell infiltration in vivo after CLMSR treatment. (**A**) Immunofluorescence analysis of tumor sections showing macrophage polarization markers. (**B**) Immunofluorescence analysis of T cell infiltration in tumor tissues. (**C**) Flow cytometric quantitative analysis of macrophage polarization and intratumoral T cell infiltration

Immunofluorescence staining indicated that CLMSR significantly upregulated CD80 expression and downregulated CD206 expression relative to the other groups (Fig. 7A, S9A and S9B). These findings suggest that in the tumor-bearing model, CLMSR effectively delivers p38 and TFEB to M2-like tumor-associated macrophages within the tumor tissues, promotes M1 polarization of macrophages, and achieves potent tumor immunotherapy. This shift in TAM profiles was associated with increased expression of T cell marker CD4 and CD8 in the CLMSR treatment group (Fig. 7B, S9C and S9D). The flow cytometry results also demonstrated a significant increase in CD80⁺ M1 macrophages accompanied by a marked reduction in CD206⁺ M2 macrophages, as well as a robust elevation of intratumoral CD8⁺ T cell infiltration, confirming the immunofluorescence observations at the single-cell level (Fig. 7C). These findings demonstrate that CLMSR effectively remodeled the tumor microenvironment by increasing M1 TAMs, CD8^+^ T cells, and CD4^+^ T cells, while reducing M2 TAMs. This shift transformed the anti-inflammatory environment into an inflammatory one.

## Discussion

TAMs are key players in the tumor microenvironment (TME), often promoting tumor growth and immune evasion. TAMs are typically characterized as M2 macrophages, activated by Th2 cytokines like IL-4 and IL-13, which promote tumor growth [36]. In contrast, M1 macrophages, activated by Th1 cytokines such as IFN-γ, are tumor-killing [37] Therefore, a more effective strategy is to convert M2 TAMs into M1 antitumor macrophages rather than depleting them [38–40]. In this study, we developed an innovative antitumor strategy using saRNA encapsulated in engineered nanoparticles to reprogram tumor-associated macrophages (TAMs) towards an M1 phenotype. The saRNAs were delivered via a metal-organic framework (MOF)-based platform and then coated with a membrane derived from exosomes expressing the TAM-targeting peptide CRV (CRVLRSGSC) and liposomes. By reprogramming TAMs to the M1 phenotype, we aimed to enhance their antitumor functions and improve therapeutic efficacy.

Previous study reveals that the role of lysosomal calcium in macrophage polarization [3]. Released lysosomal calcium not only activates p38 and NF-κB, which mediate M1 phenotype formation, but also activates TFEB, a critical regulator of lysosome biogenesis and M1-associated glycolysis. These Ca²⁺ signaling-mediated p38/NF-κB and TFEB pathways are independent but both essential for full M1 macrophage polarization. This discovery highlights an unexpected role for lysosomes in macrophage polarization and underscores the complexity of the underlying mechanisms.

Compared with cytokine-based therapies (e.g., IFN-γ or LPS), which often induce broad, non-specific inflammatory responses and systemic toxicity [41]. Moreover, unlike small-molecule drugs that may suffer from limited specificity and significant off-target effects due to their broad kinase inhibition profiles, which may unintentionally affect other immune or stromal cell populations [3, 42]. Unlike siRNA, which suppresses gene expression, saRNA selectively activates endogenous gene transcription at the promoter level, enabling a more physiological and sustained upregulation of target genes without introducing exogenous DNA or proteins [43]. This feature is particularly advantageous for macrophage polarization, as M1 reprogramming requires coordinated activation of multiple intracellular pathways rather than transient external stimulation [44]. Therefore, our approach leverages the specificity of saRNAs to upregulate genes involved in macrophage polarization.

The use of saRNAs targeting the promoters of p38 and TFEB allowed us to effectively induce the expression of these genes, promoting the transition of TAMs from the M2 to the M1 phenotype. The ratio of p38 to TFEB was optimized to 2:1 for maximal effect, based on our findings that this combination significantly upregulated M1 markers (CD80 and iNOS) and downregulated M2 markers (Arg1 and CD206).

The innovative design of our nanoparticles played a crucial role in the effectiveness of this strategy. The MOF-based core provided stability and efficient encapsulation of saRNAs. Coating this core with CRV-expressed exosome membranes facilitated targeted delivery to TAMs within the TME. Moreover, our results showed that CLMSR significantly increased the expression of M1 markers while decreasing the expression of M2 markers, confirming the successful induction of M1 polarization. In vitro experiments demonstrated that the conditioned medium from M1 macrophages induced by CLMSR significantly inhibited the proliferation, migration, and invasion of cancer cells after 48 h of incubation. This medium also suppressed the growth of 3D tumor spheroids and induced cell death within these cultures, highlighting the potent antitumor activity of CLMSR through the reprogramming of TAMs, effectively suppressing tumor growth in a controlled environment.

 In vivo studies on LLC tumor-bearing mice demonstrated the efficacy of our CLMSR nanoparticle formulation. Exosomes, known for their low immunogenicity and high stability in blood, served as ideal carriers. The use of exosome membranes (EM) to camouflage the nanoparticles improved surface functionality, reduced opsonization by blood proteins, and decreased phagocytosis by immune cells, thereby extending the circulation lifetime of the nanoparticles [21, 45]. The CLMSR treatment group exhibited significant tumor suppression compared to controls, with no notable weight loss or major organ toxicity observed. This indicates that our strategy is not only effective but also safe. Histological analyses, including HE staining and TUNEL assays, confirmed the therapeutic benefits, showing significant tumor necrosis and higher apoptotic rates in mice treated with CLMSR. Immunofluorescence staining further validated the successful reprogramming of TAMs, with increased expression of the M1 marker CD80 and decreased expression of the M2 marker CD206. Additionally, treatment with CLMSR led to an increase in the proportion of CD4 + and CD8 + T cells within the tumor microenvironment. This shift in TAM profiles and T cell infiltration is crucial, as M1 macrophages and cytotoxic T cells are associated with tumor suppression [46], while M2 macrophages promote tumor growth [47]. The antitumor effects of CLMSR may be attributed to the M1 macrophages secreting proinflammatory cytokines such as TNFα and IL1α, which stimulate T cell infiltration and activation in tumor tissue [48]. This effective reprogramming of TAMs from an M2 to an M1 phenotype, along with the increased presence of cytotoxic T cells, enhances the antitumor immune response and highlights the potential of CLMSR as a powerful therapeutic strategy for cancer treatment.

## Conclusions

In conclusion, our study presents a novel and effective macrophage immunotherapy by reprogramming TAMs to enhance antitumor immune responses, paving the way for new treatments in cancer therapy. The use of CLMSR nanoparticles offers a targeted, efficient, and safe approach to combat tumor growth and progression. Our findings reveal a surprising and critical role for lysosomal calcium in macrophage polarization, providing deeper insights into the intricate mechanisms of macrophage biology and their therapeutic implications.

## Supplementary Information

Supplementary material 1.

## Data Availability

All data generated or analyzed during this study are available from the corresponding author upon reasonable request.
