# Supplementary material for: Biomimetic proteolipid vesicles delivering small activating RNA to activate the macrophage immunotherapy for the treatment of lung cancer
Source: J Nanobiotechnology. 2026 Apr 16;24:508. doi: 10.1186/s12951-026-04392-4 (PMC13220422; doi:10.1186/s12951-026-04392-4)
Supplement: Supplementary file 1 — Supplementary material 1. [file 12951_2026_4392_MOESM1_ESM.docx]

**Supporting information**


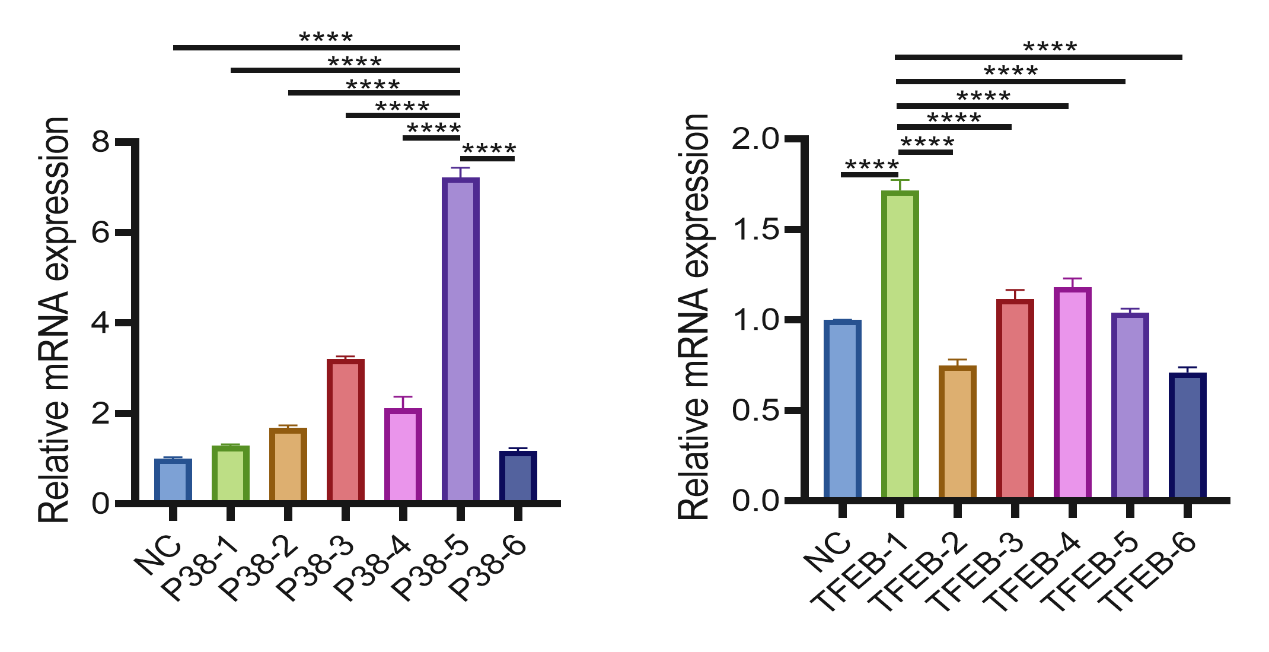


**Figure S1.** The mRNA expression of p38 and TFEB.

**
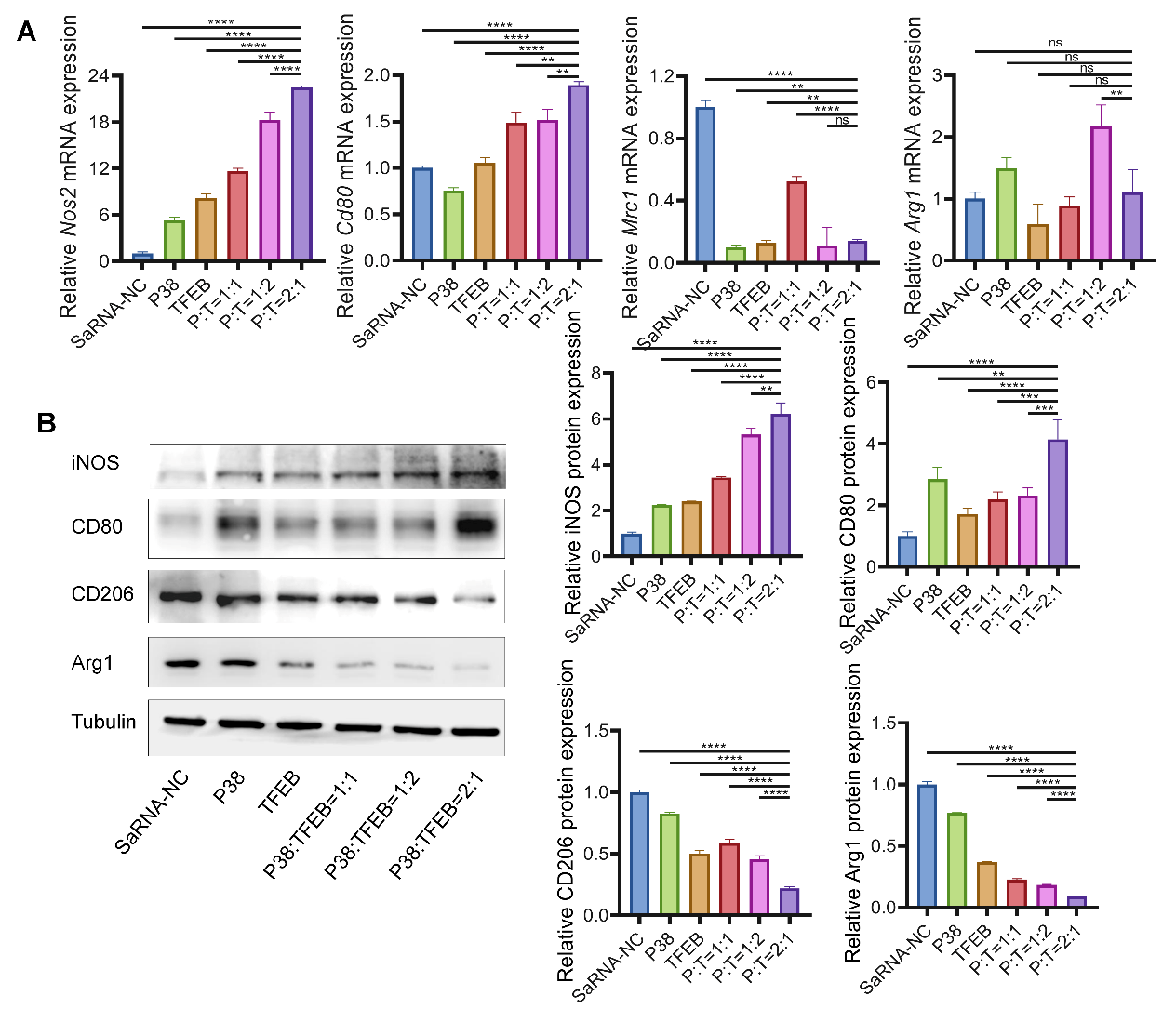
**

**Figure S2.** The influence of the ratio of p38 and TFEB on the phenotype transition of macrophages.

**
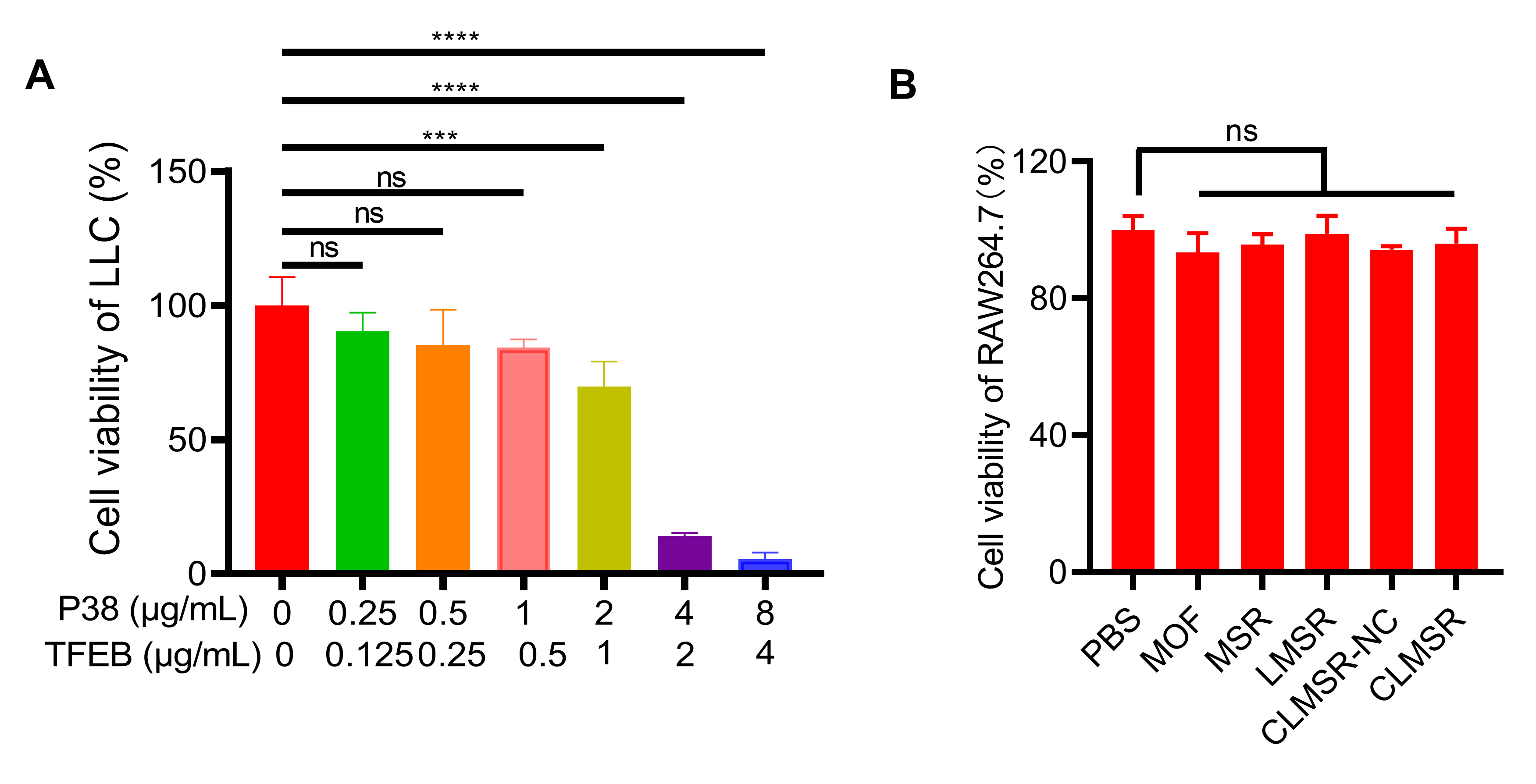
**

**Figure S3.** The cell viability assessment of p38, TFEB and MOF-based nanocarriers in RAW264.7 macrophages. All data are presented as the mean ± SD; ns, no significance.





**Figure S4.** The encapsulation efficiency of saRNA by MOF.


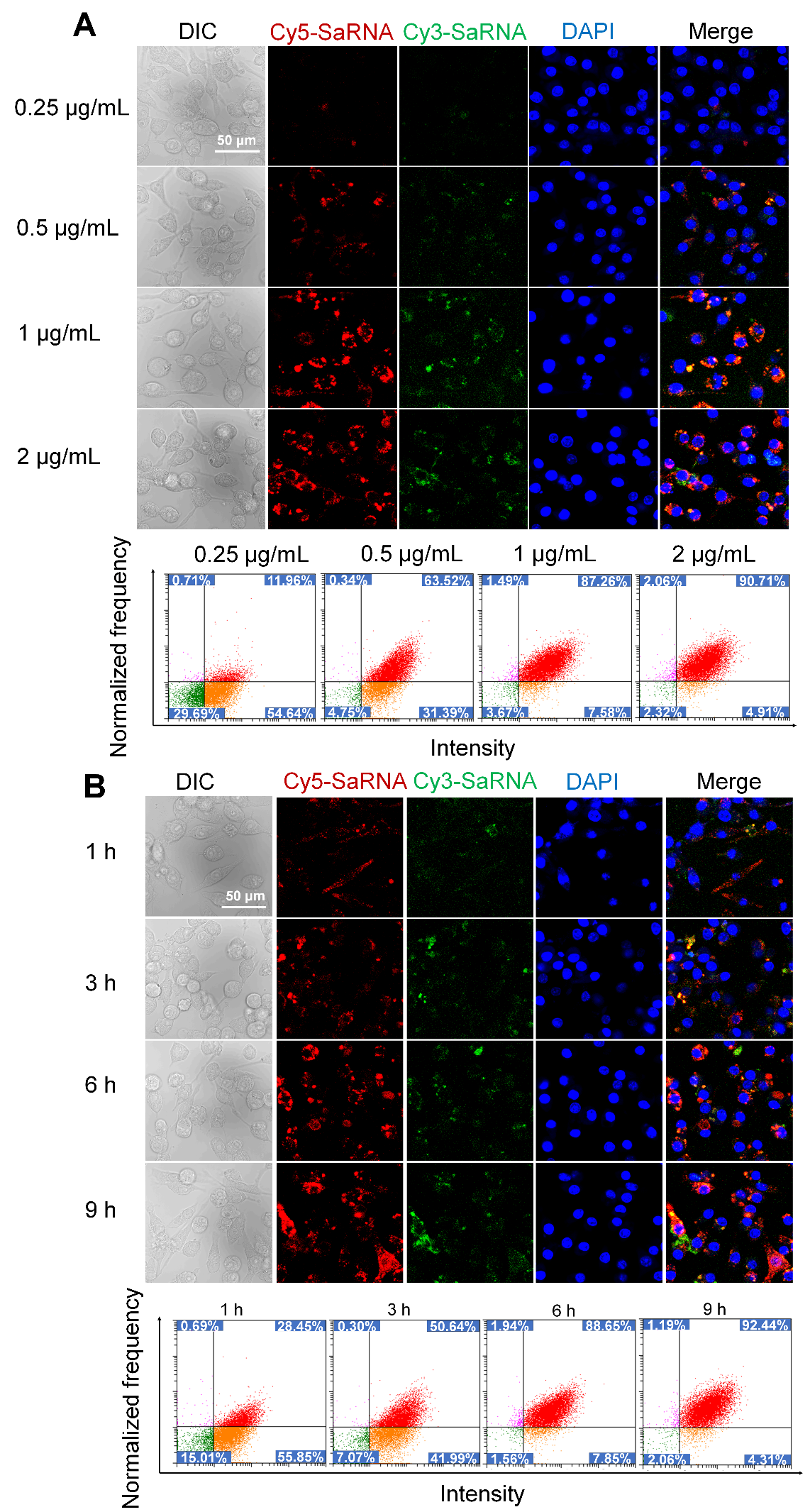


**Figure S5.** Cellular uptake of CLMSR in induced M2 macrophages observed by CLSM. (A) Transfection efficiency at varying concentrations of CLMSR. (B) Transfection efficiency at different time intervals.


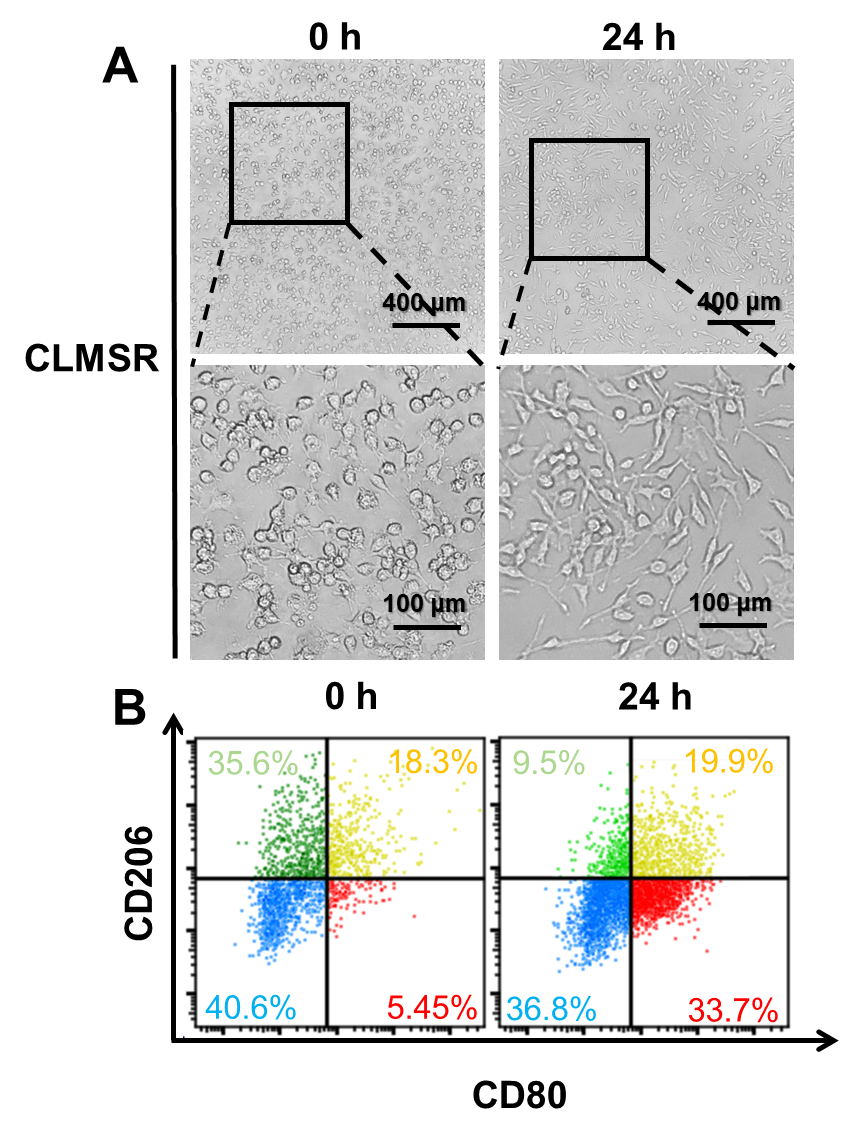


**Figure S6.** CLMSR induces M2-to-M1 macrophage reprogramming. (A) Representative bright-field images showing macrophage morphology after CLMSR treatment. (B) Flow cytometry analysis of macrophage surface marker expression following CLMSR treatment.





**Figure S7.** *In vitro* antitumor activity of CLMSR. (A) Live/dead staining, (B) cell migration and (C) cell invasion was quantitatively analyzed after treatment with PBS, CLMSR-NC, free saRNA, MSR, LMSR and CLMSR, respectively. (D) Bright-field microscopy analysis of 3D tumor spheroids under different treatments at days D2, D4, D6, D8, and D10. All data are presented as the mean ± SD; ns, no significance; ^*^*P* < 0.05, ^**^*P* < 0.01, ^***^*P* < 0.001, and ^****^*P* < 0.0001 vs treatment with CLMSR group.


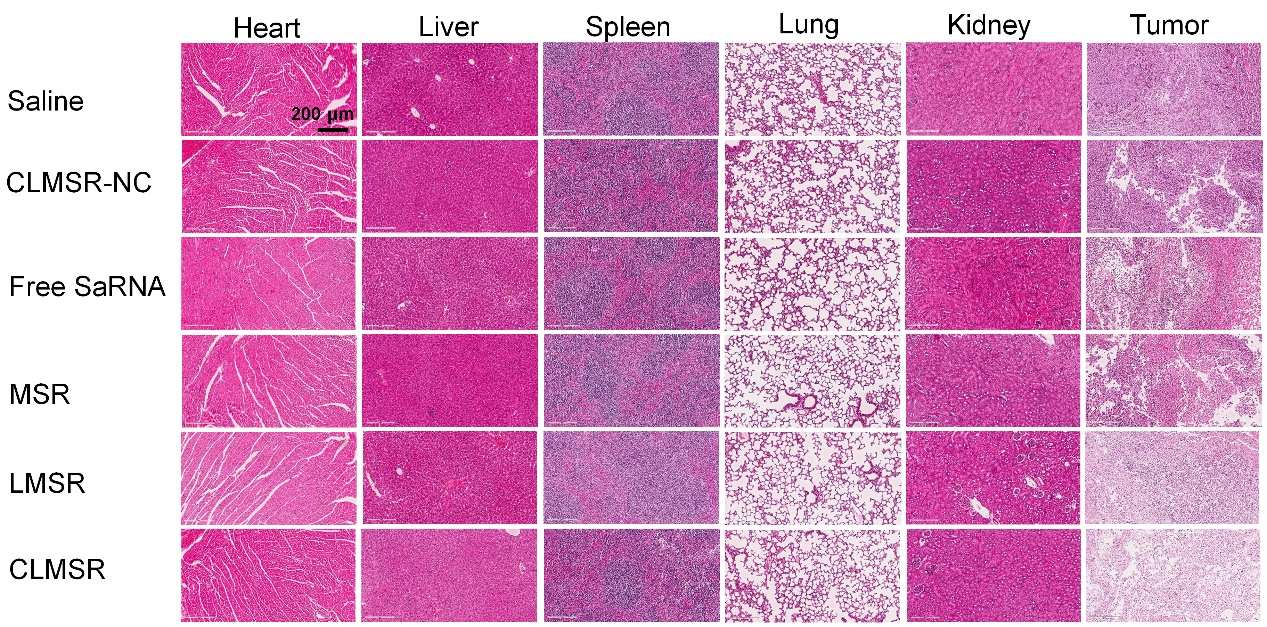


**Figure S8.** Representative H&E-stained slices of major organs and tumors.


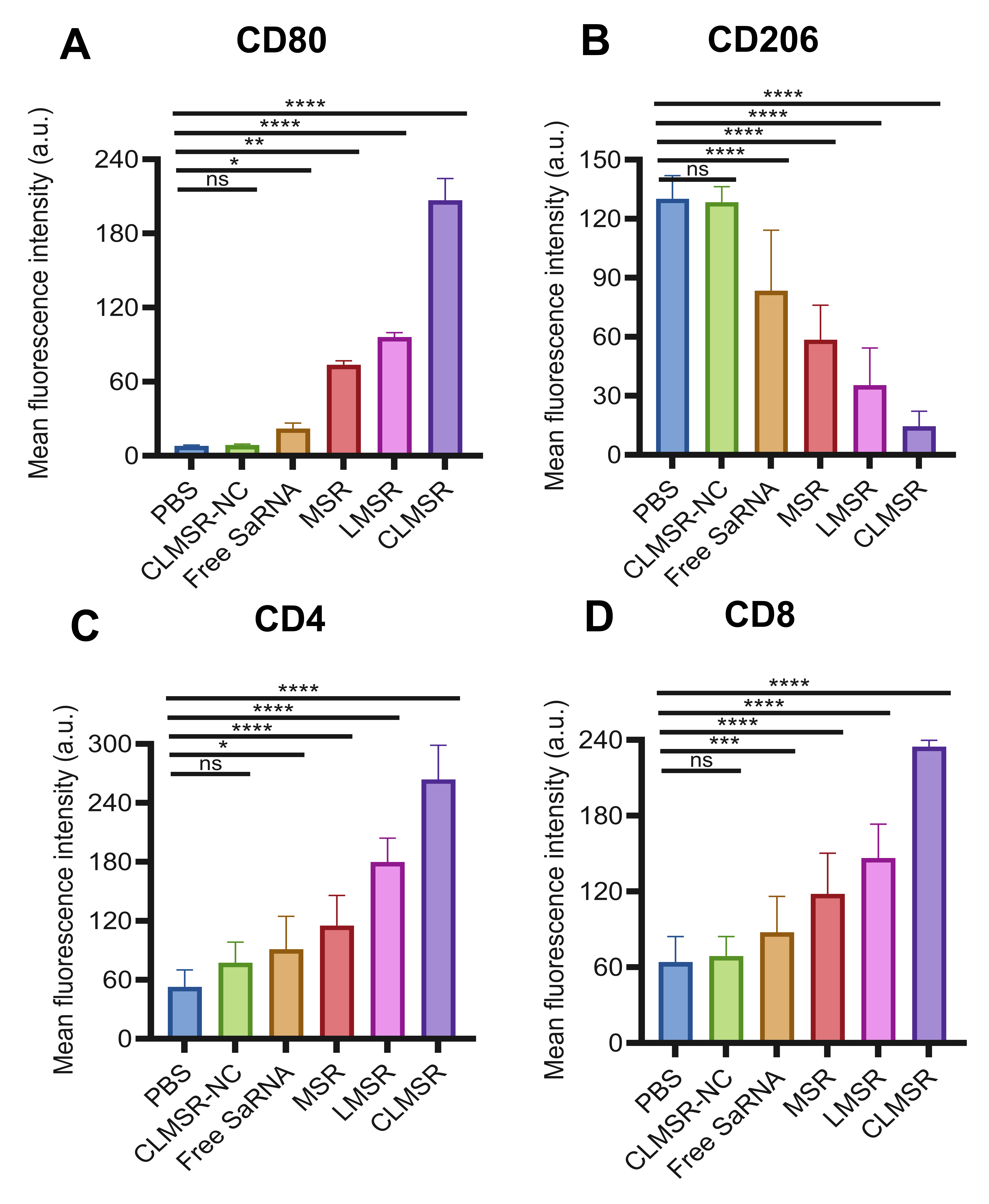


**Figure S9.** Quantitative immunofluorescence analysis of tumor tissues following treatment with various formulations.

**Table S1. Primers for RT-qPCR**

| Gene | Sequence (5'‑3') |
| --- | --- |
| *Nos2* Forward | GTTCTCAGCCCAACAATACAAGA |
| *Nos2* Reverse | GTGGACGGGTCGATGTCAC |
| *Arg1* Forward | CTCCAAGCCAAAGTCCTTAGAG |
| *Arg1* Reverse | AGGAGCTGTCATTAGGGACATC |
| *Cd80* Forward | GCAGGATACACCACTCCTCAA |
| *Cd80* Reverse | AAAGACGAATCAGCAGCACAA |
| *Mrc1* Forward | GGAGTGGCAGGTGGCTTATG |
| *Mrc1* Reverse | CACTGCTCGTAATCAGCCTCC |
| *Gapdh* Forward | CCCTCAACGACCACTTTGTC |
| *Gapdh* Reverse | AGGGGAGATTCAGTGTGGTG |
